# Impact of computerized physician order entry (CPOE) system on the outcome of critically ill adult patients: a before-after study

**DOI:** 10.1186/1472-6947-11-71

**Published:** 2011-11-19

**Authors:** Hasan M Al-Dorzi, Hani M Tamim, Antoine Cherfan, Mohamad A Hassan, Saadi Taher, Yaseen M Arabi

**Affiliations:** 1 King Abdulaziz Medical City, Riyadh, 11426, Saudi Arabia; 2 College of Medicine, King Saud bin Abdulaziz University for Health Sciences, Riyadh 11426, Saudi Arabia

**Keywords:** Intensive care unit, critical illness, CPOE, safety management, mortality, morbidity

## Abstract

**Background:**

Computerized physician order entry (CPOE) systems are recommended to improve patient safety and outcomes. However, their effectiveness has been questioned. Our objective was to evaluate the impact of CPOE implementation on the outcome of critically ill patients.

**Methods:**

This was an observational before-after study carried out in a 21-bed medical and surgical intensive care unit (ICU) of a tertiary care center. It included all patients admitted to the ICU in the 24 months pre- and 12 months post-CPOE (Misys^®^) implementation. Data were extracted from a prospectively collected ICU database and included: demographics, Acute Physiology and Chronic Health Evaluation (APACHE) II score, admission diagnosis and comorbid conditions. Outcomes compared in different pre- and post-CPOE periods included: ICU and hospital mortality, duration of mechanical ventilation, and ICU and hospital length of stay. These outcomes were also compared in selected high risk subgroups of patients (age 12-17 years, traumatic brain injury, admission diagnosis of sepsis and admission APACHE II > 23). Multivariate analysis was used to adjust for imbalances in baseline characteristics and selected clinically relevant variables.

**Results:**

There were 1638 and 898 patients admitted to the ICU in the specified pre- and post-CPOE periods, respectively (age = 52 ± 22 vs. 52 ± 21 years, p = 0.74; APACHE II = 24 ± 9 vs. 24 ± 10, p = 0.83). During these periods, there were no differences in ICU (adjusted odds ratio (aOR) 0.98, 95% confidence interval [CI] 0.7-1.3) and in hospital mortality (aOR 1.00, 95% CI 0.8-1.3). CPOE implementation was associated with similar duration of mechanical ventilation and of stay in the ICU and hospital. There was no increased mortality or stay in the high risk subgroups after CPOE implementation.

**Conclusions:**

The implementation of CPOE in an adult medical surgical ICU resulted in no improvement in patient outcomes in the immediate phase and up to 12 months after implementation.

## Background

Computerized Physician Order Entry (CPOE) is the process of entering medication orders and other physician's instructions electronically using a computer-based system to ensure standardized, legible and complete orders [1]. Its implementation has been recommended to improve patient safety and outcomes [2,3] primarily by reducing medication errors [4] that usually arise from faulty prescriptions [5]. However, CPOE itself can lead to new types of errors [6-8], which may be detrimental to patients. This may be more pronounced in critically ill patients, a more vulnerable population due to the severity of their illness, the complexity of interventions done and the diversity of equipments used in the intensive care unit (ICU). The evidence for the CPOE effectiveness in improving the outcomes of critically ill patients is scarce and remains controversial. Two before-after studies in pediatric ICUs showed no significant change in mortality after CPOE implementation [9,10]. Del Beccaro et al observed a statistically insignificant decrease in mortality from 4.2% to 3.46% in the 13-month pre- and post-CPOE implementation [9]. Keene et al found no change in mortality post-CPOE after adjusting for all covariates (OR, 0.71; 95% CI, 0.32-1.57)[10]. On the other hand, Han and colleagues observed an increase in mortality from 2.8% to 6.6% during the 5-month period after the initiation of a CPOE system at a pediatric hospital with a tertiary care ICU [11]. Literature lacks information on the impact of CPOE implementation on the outcomes of adult critically ill patients. The objective of our study, conducted as a quality improvement project, was to evaluate the outcomes of critically ill patients after CPOE implementation in an adult ICU.

## Methods

### Setting

The study was conducted at a 21-bed medical surgical closed ICU in a 900-bed teaching tertiary care hospital, King Abdulaziz Medical City-Riyadh, in Saudi Arabia. It was staffed by board certified intensivists 24 hours per days, 7 days a week as described in a study from our ICU elsewhere [12] with rotating residents from different specialties. Nursing staff were multinational with patient-to-nurse ratio of 1:1. The hospital was accredited by Joint Commission International.

### Study Design

The study was conducted as an observational before-after study. Data were extracted from a prospectively collected electronic ICU database. We included all consecutive patients admitted to the adult ICU (age ≥ 12 years) in the 24 months pre- and 12 months post-CPOE implementation. We excluded patients with brain death admitted as potential organ donors. For patients who had multiple admissions to the ICU within the same hospitalization, only the first admission was considered in the analysis. The institutional review board of King Abdulaziz Medical City approved the study and due to its observational nature, informed consent was waived.

### CPOE Implementation

Before CPOE implementation, physicians wrote medication orders on paper; nurses then faxed the orders to the ICU satellite pharmacy; the pharmacist would screen the order, make necessary adjustments after consulting with the prescribing physician if needed, transcribe the order and dispense the medication. The adult ICU was the first area in the hospital to implement CPOE. Other hospital areas continued to use the traditional paper-based order writing during the study period. The used system was a product from Misys^®^, which was later acquired by QuadraMed, and allowed medication and nonmedication orders as well as access to the patient's chart, laboratory and radiological data. It included a basic real-time clinical decision support system so that important interactions of prescribed medications, dose checking and allergy notification appeared at the time of prescription as pop-ups. In the year preceding CPOE implementation, a multidisciplinary team prepared electronic order sets and protocols. The team used the existing paper-based order sets including ICU admission orders, traumatic brain injury protocol and antimicrobial therapy order set and converted them with some modifications into CPOE order sets. Some of these order sets, such as the traumatic brain injury protocol, implemented since 2001, had been studied in our ICU and been shown to improve outcomes [13]. This was followed by training of ICU physicians, pharmacists and nurses on the use of CPOE functions. Following the "Go Live" on December 4, 2006, physicians directly entered all medications and most nonmedication orders, such as specific nursing instructions, requisition of laboratory tests and radiological examinations and consultations of physiotherapy and nutrition services, from any computer terminal inside or outside the unit. Pop-up alerts appeared on the screen and required verification before order acceptance. Pharmacists screened electronic prescriptions and advised physicians in case of errors. Nurses checked their bedside computer terminals for new and scheduled orders. Some orders, such as diet and blood transfusions, continued on papers. Technical support and guidance were provided 24 hours a day, 7 days a week for 2 weeks from the hospital's information technology specialists, then as needed. To address the observed difficulties and limitations and enhance the CPOE system, orders and order sets were revised upon agreement of physicians, nurses, clinical pharmacists and medical informatics specialists. Such revisions included transforming complex intravenous orders that required more than 3 steps into single-step orders, optimizing doses and frequency of intravenous antibiotics and simplifying radiological and laboratory orders.

### Measurements and outcomes

The following data were extracted from the ICU database: age, gender, Acute Physiology and Chronic Health Evaluation (APACHE) II score [14], source of admission (ward, emergency department, operating room and other hospital), main reason for ICU admission (as per APACHE II definitions), cardiac arrest and sepsis as a reason for admission, the presence of chronic health illness (as defined by APACHE II system), admission Glasgow Coma Scale, admission creatinine and International Normalized Ratio (INR), requirement for vasopressors within the first 24 hours after admission and requirement for mechanical ventilation during ICU stay. The primary outcome was ICU mortality. Secondary outcomes were: duration of mechanical ventilation, ICU and hospital length of stay and hospital mortality. Comparisons were made for different time periods: one, three and 24 months before implementation compared with one, three and 12 months post-implementation. The reason for selecting these periods was to evaluate CPOE effect in the immediate and more extended period of implementation and also to control possible seasonal variation of different outcomes. In addition, we evaluated the impact of CPOE in selected groups of patients, whose outcome was thought to be particularly affected by CPOE. These included: age 12-17 years since Han et al reported an increased mortality in the pediatric patients post-CPOE implementation [11], diagnosis of sepsis because outcomes of these patients can be affected by timely interventions such as timely fluid and antimicrobial therapy [15] and traumatic brain injury as such patients may benefit from protocolized management. Additionally, because patients with higher severity of illness were thought to be more vulnerable, we evaluated the effect of CPOE in patients with high APACHE II score,≥ the median score which was 23.

### Statistical analysis

SAS software (version 9.0; SAS Institute, Cary, NC) was used to analyze data. Continuous variables were reported as means with standard deviation and categorical variables as absolute and relative frequencies. The Chi-squared test was used to assess the differences between categorical variables and the student t-test was used for the analysis of differences in the means of continuous variables. To examine the association between CPOE implementation and various outcomes, we used stepwise multivariate logistic regression analysis. Independent variables entered in the model were important factors that clinically affect outcomes and baselines characteristics that were significantly different in the 24 month pre- and 12 months post-CPOE in addition to CPOE implementation status. The following variables were entered in the model: age, gender, APACHE II, source of admission, non-operative trauma and post-operative trauma as main reason for admission, cardiac arrest as a reason for admission, chronic health illness (liver, cardiovascular and renal), creatinine, INR, vasopressors use, and mechanical ventilation. Results were reported as adjusted odds ratios (aOR) for categorical outcomes (ICU and hospital mortality) and beta (β) coefficient for continuous outcomes (duration of mechanical ventilation, length of stay in the ICU and the hospital) with 95% confidence intervals (CI). This analysis was performed for the whole cohort and for the selected subgroups of patients. At α = 0.05, our study had an 80% power to detect a 4.8% decrease or a 5.2% increase in mortality after CPOE implementation using the observed mortality (23.4%) in pre-CPOE implementation period and the number of patients in the pre-CPOE (n = 1638) and post-CPOE (n = 898) periods. A P-value < 0.05 was considered statistically significant.

## Results

### General characteristics of all patients

In the 24 months pre-CPOE implementation (December 4, 2004 to December 3, 2006), 1638 patients were admitted to the ICU (13104 patient-days) compared to 898 patients (7274 patient-days) in the 12 months post-CPOE implementation (December 4, 2006 to December 3, 2007). Table 1 describes the characteristics of patients admitted to the ICU in the following periods: 24 vs. 12 months, 12 vs. 12 months, 3 vs. 3 months and 1 vs. 1 month pre- and post-CPOE implementation. There were no significant differences in age, admission APACHE II score, admission Glasgow Coma Scale, creatinine and INR in these pre- and post-implementation periods. Compared to the 24 months pre-CPOE implementation, the percentage of female patients and postoperative trauma admissions were higher in the following 12 months (39% vs. 35%, p = 0.04). Also there were significant imbalances between the two groups in these variables: source of admission before ICU, chronic health illnesses and cardiac arrest as a reason for ICU admission. In addition, vasopressors (50% vs. 36%, p < 0.0001) and mechanical ventilation (77% vs. 73%, p = 0.004) were used more commonly in the 24 months pre-CPOE compared to 12 month post-CPOE implementation.

**Table 1:** Comparison of baseline characteristics of admissions to the intensive care unit pre- and post-computerized physician order entry (CPOE) implementation in four different periods: 24 months pre- vs. 12 months post-CPOE, 12 months pre- vs. 12 months post-CPOE, three months pre- vs. three months post-CPOE vs. one month pre- and one month post-CPOE.

Variable	24 months pre N = 1638	12 months post N = 898	P-value	12 months pre N = 813	12 months post N = 898	P-value	3-months pre N = 192	3-months post N = 239	P-value	1-month pre N = 69	1-month post N = 75	P-value
Age in years, mean ± SD	52.1 ± 21.5	52.4 ± 21.1	0.74	51.7 ± 21.7	52.4 ± 21.1	0.51	50.1 ± 21	53.6 ± 21.1	0.09	48.0 ± 22.2	50.3 ± 21.0	0.52
Female gender, N (%)	567 (34.7)	349 (38.9)	0.04	284 (34.9)	349 (38.9)	0.09	64 (33.3)	97 (40.6)	0.12	28 (40.6)	32 (42.7)	0.80
APACHE II, mean ± SD	24.1 ± 9.4	24.2 ± 9.7	0.83	24.7 ± 9.6	24.2 ± 9.7	0.36	25.1 ± 10.2	25.1 ± 10.4	1.0	25.3 ± 9.3	24.7 ± 10.9	0.75
Source of admission, N (%)												
Emergency department	450 (27.5)	179 (19.9)		223 (27.4)	179 (19.9)		64 (33.3)	50 (20.9)		25 (36.2)	22 (29.3)	
Ward	668 40.8)	331 (36.9)	0.0001	326 (40.1)	331 (36.9)	< 0.0001	75 (39.1)	101(42.3)	0.02	28 (40.6)	22 (29.3)	0.14
Operating room	418 (25.5)	335 (39.8)		214 (26.3)	335 (39.8)		43 (22.4)	73 (30.5)		14 (20.3)	27 (36.0)	
Other hospital	100 (6.1)	53 (5.9)		43 (5.3)	53 (5.9)		10 (5.2)	15 (6.3)		2 (2.9)	4 (5.3)	
Main reason for admission, N (%)												
Respiratory	304 (18.6)	150 (16.7)	0.24	136 (16.7)	150 (16.7)	0.99	44 (22.9)	46 (19.3)	0.35	22 (31.9)	16 (21.3)	0.15
Cardiovascular	514 (31.4)	258 (28.7)	0.16	269 (33.1)	258 (28.7)	0.05	47 (24.5)	70 (29.3)	0.26	11 (15.9)	21 (28)	0.08
Neurologic	119 (7.3)	66 (7.4)	0.94	60 (7.4)	66 (7.4)	0.98	22 (11.5)	23 (9.6)	0.54	10 (14.5)	2 (2.7)	0.01
Other medical	83 (5.1)	35 (3.9)	0.18	35 (4.3)	35 (3.9)	0.67	7 (3.7)	8 (3.4)	0.87	3 (4.4)	1 (1.3)	0.27
Non-operative trauma	229 (14)	83 (9.2)	0.0005	108 (13.3)	83 (9.2)	0.008	30 (15.6)	23 (9.6)	0.06	9 (13)	9 (12)	0.85
Post-operative trauma	387 (23.7)	306 (34.1)	< 0.0001	205(25.2)	306 (34.1)	< 0.0001	42 (21.9)	69 (28.9)	0.10	14 (20.3)	26 (34.7)	0.05
Cardiac arrest as admission diagnosis	135 (8.3)	45 (5.0)	0.002	76 (9.4)	45 (5.0)	0.0005	17 (8.9)	13 (5.4)	0.17	6(8.7)	2 (2.7)	0.11
Sepsis on admission	420 (25.7)	251 (28.0)	0.21	241 (29.6)	251 (28.0)	0.21	51 (26.6)	67 (28.0)	0.73	16 (23.2)	19 (25.3)	0.76
Chronic health illnesses, N (%)												
Chronic liver disease	259 (15.8)	97 (10.8)	0.0005	117 (14.4)	97 (10.8)	0.03	26 (13.5)	32 (13.4)	0.96	10 (14.5)	7 (9.3)	0.34
Chronic cardiovascular	451 (27.6)	289 (32.2)	0.01	250 (30.8)	289 (32.2)	0.52	59 (30.7)	114 (47.7)	0.0004	24 (34.8)	31 (41.3)	0.42
Chronic respiratory	413 (25.2)	224 (24.9)	0.87	259 (31.9)	224 (24.9)	0.0015	57 (29.7)	94 (39.3)	0.04	27 (39.1)	26 (34.7)	0.58
Chronic renal disease	349 (21.3)	157 (17.5)	0.02	210 (25.8)	157 (17.5)	< .0001	54 (28.1)	64 926.8)	0.76	19 (27.5)	23 (30.7)	0.68
Immunocompromised	228 (13.9)	124 (13.8)	0.93	117 (14.4)	124 (13.8)	0.93	19 (9.9)	42 (17.6)	0.02	7 (10.1)	11 (14.7)	0.41
Glasgow coma scale	9.2 ± 4.3	8.9 ± 4.5	0.17	8.7 ± 4.5	9.2 ± 4.3	0.01	8.4 ± 4.4	9.0 ± 4.2	0.15	8.4 ± 4.0	9.3 ± 4.0	0.22
Admission creatinine in μmol/L	163 ± 161	158 ± 146	0.44	166 ± 164	158 ± 146	0.37	195 ± 214	162 ± 142	0.08	162 ± 170	167 ± 165	0.87
Admission INR	1.6 ± 1.1	1.5 ± 0.8	0.10	1.6 ± 1.0	1.5 ± 0.8	0.24	1.5 ± 0.8	1.4 ± 1.0	0.57	1.4 ± 0.8	1.8 ± 1.5	0.09
Vasopressor use*	822 (50.2)	327 (36.4)	< 0.0001	373 (45.9)	327 (36.4)	< 0.0001	92 (47.9)	97 (40.6)	0.13	28 (40.6)	33 (44.0)	0.68
Mechanically ventilated, No.%	1265 (77.3)	662 (73.7)	0.04	636 (78.2)	662 (73.7)	0.03	152 (79.2)	179 (74.9)	0.29	55 (79.7)	62 (82.7)	0.65

ICU: intensive care unit; CPOE: computerized physician order entry; APACHE: Acute Physiology and Chronic Health Evaluation; INR: International Normalized Ratio.

* Vasopressors use was defined as use of dopamine > 5 mcg/Kg/min or any dose of norepinephrine, epinephrine, phenylephrine or vasopressin.

### Outcomes of patients pre- and post-CPOE implementation

Table 2 compares the outcomes of all patients in the cohort in relationship to CPOE implementation. The implementation of CPOE was not associated with a statistically significant change in ICU mortality when comparing the 24 months pre-CPOE to the 12 months post-CPOE (aOR 0.98, 95% CI 0.7-1.3), the 12 months pre-CPOE to the 12 months post-CPOE (aOR 0.96, 95% CI 0.7-1.3), the 3 months pre-CPOE to the 3 months post-CPOE (aOR 0.83, 95% CI 0.5-1.5), the 1 month pre-CPOE to the 1 month post-CPOE implementation (aOR 1.17, 95% CI 0.4-3.2). Similarly, CPOE implementation was not associated with significant differences in hospital mortality, duration of mechanical ventilation and of length of stay in the ICU and hospital in any of the four periods.

**Table 2:** Outcomes of patients in different pre- and post-computerized physician order entry (CPOE) periods with adjusted risk calculated using multivariate logistic regression analysis.

A. Comparisons in the 24 months pre- vs. 12 months post-CPOE implementation					
**Variable**	**Pre-CPOE**	**Post-CPOE**	**Adjusted OR*^a ^*or β*^b ^*coefficient**	**95% CI**	**P-value**
ICU mortality, N (%)	382 (23.4)	187 (20.8)	0.98	0.7 - 1.3	0.87
Hospital mortality, N (%)	633 (38.7)	319 (35.5)	1.00	0.8 - 1.3	0.80
Mechanical ventilation duration in days, mean ± SD	7.2 ± 9.8	6.3 ± 10.8	-0.30	-1.3 - 0.7	0.54
ICU LOS in days, mean ± SD	8.0 ± 10.5	8.1 ± 16.9	1.13	-0.2 - 2.5	0.10
Hospital LOS in days, mean ± SD	46.8 ± 77.5	46.6 ± 84.6	3.60	-4.5 - 11.8	0.39

**B. Comparisons in the 12 months pre- vs. 12 months post-CPOE implementation**					

Variable	Pre-CPOE	Post-CPOE	Adjusted OR*^a ^*or β*^b ^*coefficient	95% CI	P-value
ICU mortality, No.%	190 (23.4)	187 (20.8)	0.96	0.7 - 1.3	0.78
Hospital mortality, No.%	296 (36.4)	319 (35.5)	1.24	0.9 - 1.6	0.13
MV duration, mean ± SD	7.6 ± 10.3	6.3 ± 10.8	-0.56	-1.7 - 0.6	0.35
ICU LOS, mean ± SD	8.4 ± 10.5	8.1 ± 16.9	0.78	-0.9 - 2.5	0.37
Hospital LOS, mean ± SD	44.2 ± 64.5	46.6 ± 84.6	5.04	-4.1 - 14.2	0.28

**C. Comparisons in the 3 months pre- vs. 3 months post-CPOE implementation**					

Variable	Pre-CPOE	Post-CPOE	Adjusted OR*^a ^*or β*^b ^*coefficient	95% CI	P-value
ICU mortality, N (%)	52 (27.1)	55 (23.0)	0.83	0.5 - 1.5	0.55
Hospital mortality, N (%)	74 (38.5)	90 (37.7)	1.04	0.6 - 1.8	0.90
Mechanical ventilation duration in days, mean ± SD	8.0 ± 9.9	6.1 ± 8.0	-1.49	-3.2 - 0.2	0.09
ICU LOS in days, mean ± SD	9.2 ± 11.2	6.9 ± 8.4	-1.77	-3.6 - 0.1	0.06
Hospital LOS in days, mean ± SD	41.2 ± 53.9	41.1 ± 53.1	1.21	-10.4 - 12.8	0.84

**D. Comparisons in the 1 month pre- vs. 1 month post-CPOE implementation**					

Variable	Pre-CPOE	Post-CPOE	Adjusted OR*^a ^*or β*^b ^*coefficient	95% CI	P-value
ICU mortality, N (%)	16 (23.2)	16 (21.3)	1.17	0.4 - 3.2	0.77
Hospital mortality, N (%)	24 (34.8)	28 (37.3)	2.10	0.8 - 5.5	0.14
Mechanical ventilation duration in days, mean ± SD	8.3 ± 9.8	5.8 ± 9.8	-2.74	-5.4 - (-0.1)	0.04
ICU LOS in days, mean ± SD	9.1 ± 10.0	6.5 ± 6.7	-2.52	-5.3 - 0.3	0.08
Hospital LOS in days, mean ± SD	39.0 ± 45.9	41.2 ± 55.1	-1.28	-19.2 - 16.6	0.89

These outcomes include ICU and hospital mortality, duration of mechanical ventilation and length of stay in the hospital and the intensive care unit. Adjustment was done for age, gender, Acute Physiology and Chronic Health Evaluation II score, source of admission, reason for admission (non-operative trauma and post-operative trauma), cardiac arrest as a reason for admission, chronic health illnesses (liver, cardiovascular and renal), creatinine, INR, use of vasopressors, and mechanical ventilation.

ICU: intensive care unit; CPOE: computerized physician order entry; CCRT: continuous renal replacement therapy; OR: odds ratio; CI: confidence interval CPOE: computerized physician order entry, ICU: intensive care unit, LOS: length of stay, SD: standard deviation, APACHE: Acute Physiology and Chronic Health Evaluation

*a: *OR is reported for categorical outcomes.

*b: *β coefficient is reported for continuous variables.

*The other variables that remained significant in the stepwise logistic multivariate regression analysis were: male gender (OR 1.39, 95% CI 1.07-1.81), APACHE II (OR 1.09, 95% CI 1.07-1.11), requirement for mechanical ventilation (OR 21.9, 95% CI 7.62-62.94), liver cirrhosis (OR 2.97, 95% CI 2.16-4.10), postoperative trauma (OR 0.38, 95% CI 0.25-0.58) and INR (OR 1.53, 95% CI 1.34-1.76).

**The other variables that remained significant in the stepwise multivariate logistic regression analysis were: age (OR 1.01, 95% CI 1.01-1.02), APACHE II (OR 1.09, 95% CI 1.07-1.11), requirement for mechanical ventilation (OR 2.451, 95% CI 1.67-3.59), liver cirrhosis (OR 2.39, 95% CI 1.72-3.31) chronic renal failure (OR 1.61, 95% CI 1.16-2.22), postoperative trauma (OR 0.43, 95% CI 0.31-0.59), non-operative trauma (OR 0.66, 95% CI 0.44-1.01), INR (OR 1.56, 95% CI 1.34-1.82) and creatinine (OR 1.00, 95% CI 0.99-1.00).

### Outcomes of patient subgroups according to CPOE status

Table 3 describes the outcomes of four subgroups of patients in the 24 months pre- and 12 months post-CPOE implementation. There were no significant differences in ICU mortality of patients 12 to 17 years old, those with sepsis as an admission diagnosis, those with traumatic brain injury and those with APACHE II score ≥ 23.0. Also there were no significant differences in hospital mortality, the duration of mechanical ventilation and of stay in the ICU and hospital within the same periods pre- and post-CPOE in the 4 subgroups.

**Table 3:** Outcomes of four patient subgroups in the 24 months pre- and 12 months post-CPOE implementation with adjusted risk calculated using multivariate logistic regression analysis.

Variable	Pre-CPOE No. (%)	Post-CPOE No. (%)	Adjusted OR*^a ^*or β*^b ^*coefficient	95% CI	P value
**A. patients in the age group 12-17 years, N = 129**					

ICU mortality, No.%	9 (11)	5 (10.6)	0.78	0.1 - 4.1	0.77
Hospital mortality, No.%	14 (17.1)	7 (14.9)	0.93	0.3 - 3.2	0.90
Mechanical ventilation duration in days, mean ± SD	7.7 ± 7.2	6.3 ± 9.0	-0.19	-3.2 - 2.8	0.90
ICU LOS in days, mean ± SD	8.2 ± 7.2	8.6 ± 17.0	2.41	-2.6 - 7.4	0.34
Hospital LOS in days, mean ± SD	42.8 ± 59.4	31.6 ± 44.1	-6.70	-30.8 - 17.4	0.58

**B. Admission diagnosis of sepsis, N = 638**					

ICU mortality, No.%	152 (39)	96 (40.3)	1.09	0.7 - 1.7	0.70
Hospital mortality, No.%	230 (59)	141 (59.2)	1.01	0.7 - 1.6	0.96
Mechanical ventilation duration in days, mean ± SD	8.4 ± 10.6	7.3 ± 8.1	-1.62	-3.3 - 0.1	0.06
ICU LOS in days, mean ± SD	9.4 ± 10.7	8.4 ± 8.7	-1.41	-3.2 - 0.4	0.13
Hospital LOS in days, mean ± SD	40.7 ± 75.6	50.1 ± 125	13.07	-5.2 - 31.3	0.16

**C. Traumatic brain injury, N = 322**					

ICU mortality, No.%	34 (14.5)	6 (6.7)	0.53	0.1 - 1.1	0.06
Hospital mortality, No.%	44 (19)	10 (11.1)	0.62	0.3 - 1.5	0.28
Mechanical ventilation duration in days, mean ± SD	10.7 ± 7.9	9.8 ± 9.6	-0.69	-2.7 - 1.3	0.50
ICU LOS in days, mean ± SD	11.2 ± 8.2	12.8 ± 15.1	2.24	-0.3 - 4.8	0.09
Hospital LOS in days, mean ± SD	72.2 ± 118.7	72.2 ± 82.2	13.7	-14.2 - 41.6	0.33

**D. APACHE II > 23, N = 1065**					

ICU mortality, No.%	256 (35.6)	121 (35)	1.03	0.8 - 1.4	0.87
Hospital mortality, No.%	419 (58.3)	197 (56.9)	1.00	0.7 - 1.3	1.00
Mechanical ventilation duration in days, mean ± SD	10.1 ± 10.9	10.4 ± 14.1	0.02	-1.5 - 1.5	0.98
ICU LOS in days, mean ± SD	10.9 ± 11.3	11.7 ± 14.8	0.38	-1.2 - 2.0	0.64
Hospital LOS in days, mean ± SD	49.6 ± 88.2	52.2 ± 110.8	5.02	-7.2 - 17.3	0.42

These outcomes include ICU and hospital mortality, duration of mechanical ventilation and duration of stay in the hospital and the intensive care unit. Adjustment was done for age, gender, APACHE II, source of admission, reason for admission (non-operative trauma and post-operative trauma), cardiac arrest as a reason for admission, chronic health illnesses (liver, cardiovascular and renal), creatinine, INR, use of vasopressors, and mechanical ventilation.

ICU: intensive care unit; CPOE: computerized physician order entry; OR: odds ratio; CI: confidence interval; APACHE: Acute Physiology and Chronic Health Evaluation CPOE: computerized physician order entry, LOS: length of stay, SD: standard deviation.

*a: *OR is reported for categorical outcomes.

*b: *β coefficient is reported for continuous variables.

## Discussion

Our study demonstrates that CPOE implementation was not associated with any significant improvement in mortality or any change in other outcomes within 12 months of implementation in critically ill patients older than 12 years.

CPOE has been advocated as a tool to enhance patient care and safety and improve resource utilization by ensuring standardized, legible, and complete orders thus eliminating prescription errors, providing advisories orders for drugs that can potentially harm patients and offering physicians evidence-based clinical decision support. Several hospital wide studies showed benefits attributed to CPOE [16-18]. A systematic review that evaluated the effect of CPOE on patient safety found that 23 of 25 selected studies showed 13-99% relative risk reduction in medication errors, 6 of 9 selected studies showed 35-98% relative risk reduction in potential adverse drug events and 4 of 7 selected studies showed 30-84% relative risk reduction in adverse drug events [4]. Other studies showed that the impact of CPOE implementation might extend to mortality benefit. A cross-sectional study of urban hospitals in Texas found that automated order entry was associated with significant reduction in the adjusted odds of death for myocardial infarction and coronary artery bypass graft procedures [19]. Longhurst and colleagues found a decrease in the mean monthly adjusted mortality rate by 20% (95% CI 0.8%-40%, p = 0.03) at a tertiary care children hospital post-CPOE implementation [20]. Moreover, other studies showed a CPOE-associated improvement in hospital resource utilization, resulting in decreased hospital lengths of stay and costs [21,22].

On the other hand, other hospital-wide reports raised concerns about the effectiveness of CPOE in averting medication errors and promoting patient safety. In fact, some studies demonstrated that CPOE can induce errors. Koppel et al identified 22 situations in which the CPOE system facilitated medication errors [7]. These situations fell into two categories: information errors generated by fragmentation of data and flaws in human-machine interface [7]. The causes of CPOE pitfalls include poor quality of CPOE system, inadequate technical support, healthcare provider resistance to change, insufficient preparation and insufficient user training [23].

Data on CPOE implementation in ICU setting are scarce. On one hand, ICUs environment is well controlled and monitored and has higher staffing level than other areas of the hospital. On the other hand, critically ill patients are a vulnerable population and the healthcare providers working in the ICU have many responsibilities and frequently perform multiple and complex tasks and so are prone to errors. One study looked at the frequency of distraction or interruptions that occur during computerized order entry in one medical ICU and found that one distraction or interruption occurred approximately every 5 minutes and preceded 2 order entry errors [24]. Reports on the impact of CPOE on the outcome of ICU patients have revealed conflicting results. Some studies showed benefit or no harm from CPOE use in the ICU. A prospective trial that compared paper-based order entry in 14 ICU beds (80 patient-days) to CPOE implemented in 8 ICU beds (80 patient-days) found that CPOE was associated with significant lower incidence of medication prescription errors and adverse drug events [25]. Another study showed that CPOE in the ICU was associated with significant reduction in the time between ordering and reporting stat tests [26]. Development of evidence-based decision algorithm for red cell transfusion facilitated by CPOE led to significant reduction in inappropriate transfusions and decrease in blood transfusion from 1.08 ± 2.3 units 0.86 ± 2.3 units (p < 0.001)[27]. In a before-after study at a 20-bed tertiary pediatric ICU, Del Beccaro, et al observed a statistically insignificant decrease in mortality from 4.2% to 3.46% in the 13-month pre- and post-CPOE implementation respectively [9]. Keene et al also found no increased mortality post-CPOE initiation even post-adjusting for all covariates (OR, 0.71; 95% CI, 0.32-1.57) [9,10]. On the other hand, the report of Han et al [11] in 2005 was alarming. The authors reviewed the mortality data at a pediatric hospital in the 13 months before (n = 1394, 56.7% were admitted to ICU) and 5 months after (n = 548, 56.9% were admitted to ICU) implementation of a different CPOE system (Millenium Powerchart software system, Cerner Corporation, Kansas City, MO) and found a significant increase in mortality post-CPOE implementation from 2.8% to 6.6% (OR: 3.71; 95% CI: 2.13-6.46)[11]. Our study demonstrated no change in the mortality of critically ill patients > 12 years of age post-CPOE implementation suggesting safety of its implementation in our setting.

The observed differences in the outcomes among studies are probably related to inherent methodological differences, patient population as well as differences in the CPOE systems and their implementation process. Additionally, the observed impact of CPOE is influenced by the comparison group or period; as such no benefit will be observed if the CPOE is compared to a very well organized and robust paper-based order system, while the same CPOE will reveal major improvement if implemented where the paper-based system was not well established. This may explain the lack of benefit in our ICU, since the implementation converted pre-existing well established paper-based order sets into CPOE order sets. In particular, our evidence-based traumatic brain injury order set has been shown to improve outcome; hence the lack of benefit of CPOE in this population. Factors related to CPOE system and implementation also greatly influence its impact. CPOE systems vary considerably in their ease to use, ability to customize and the presence and level of clinical decision support. The success of implementation depends on multiple factors including organizational readiness for change, engagement of end users, the level of technical support and the project management. Hence, commitment of hospital management and healthcare providers to CPOE is crucial [28,29]. Sittig et al's commentary [30] on the study by Han et al [11] illustrates many of the above points. They thought that the increased mortality was related to multiple factors that accompanied CPOE implementation and significantly affected hospital workflow. These factors included quick hospital-wide implementation of the system over a 6-day period, pharmacy centralization for all medications including vasoactive agents which may have affected the care of ICU patients, delay in patient registration causing delays in management of seriously ill patients, increased physician workload leading to less focus on patient stabilization and management at presentation, wireless networking which led to delays in order entry during peak operational periods, order entering taking long time (1-2 minutes for a single order), decreased collaborative patient care due to the diminution of face-to face communication and interaction between nurses and physicians, ICU nurses spending significant amount of time at a computer terminal away from the bedside, and not using order sets [30]. Harrison and colleagues evaluated the unintended consequences of health information technology in general including CPOE and demonstrated other important factors such as the elimination of informal interactions and redundant checks that help detect errors, spending more time on order entry rather patient care, overdependence on decision support system for appropriate prescribing leading to management difficulties when CPOE was unavailable or down [31]. Additionally, Karsh et al described eleven fallacies of health information technology which undermine its efficacy and suggested that collaboration between human factors engineers, applied psychologists, medical sociologists, communication scientists, cognitive scientists and interaction designers were needed to overcome these challenges [32]. In our ICU, CPOE implementation was well planned and closely monitored. Nevertheless, we faced multiple challenges which included occasional server slowness, sporadic resistance to CPOE use, alert fatigue largely due to redundant low risk pop-ups and complex orders. That ICU was the only hospital area that implemented CPOE in the study period was another challenge that necessitated additional training efforts for residents who periodically rotated in the ICU. All of these factors may have contributed to our findings of no difference in outcomes, but subsequently led to the formation of the ICU CPOE committee to enhance CPOE and optimize orders and ordering process. From our experience, these CPOE challenges decreased with time, which suggests that CPOE effectiveness in improving outcomes may require time longer than one year to be observed. It is also important to note that some of the challenges observed in other studies were institution-specific. For example, the observed delay in the registration of patients transferred from other hospitals in Han's et al study [11] was not an issue in our hospital as the registration process did not change post-CPOE implementation. Moreover, a more refined clinical decision support system that would advise on medication dose and duration or on optimal laboratory testing rates, that would utilize patient data and provide guideline-based care recommendations, or that would aid in weaning mechanical ventilation [33] might result in different outcomes.

This study must be interpreted in light of its strengths and limitations. The study used a prospectively collected data and was the first to address the relationship between CPOE and patient outcomes in adult ICU setting. It evaluated a commercial CPOE system which may strengthen the generalizability of our findings. Moreover, we described the CPOE experience at a Saudi tertiary care ICU staffed by multi-national healthcare workers with different backgrounds in information technology. Additionally, the study was done in a period during which there was no change in the ICU structure and function. Among the limitations was the potential time bias considering the pre-post design. However, we adjusted for all potential confounders in our multivariate analysis. Because of the study design, we were not able to examine intermediate endpoints such as technical performance, adverse drug events, adherence to guidelines and staff satisfaction. We believe that these endpoints should be targets for future research.

## Conclusions

In conclusion, our study found that CPOE implementation in an adult medical surgical ICU was associated with no improvement in ICU and hospital mortality in the immediate period and up to 12 months after implementation. CPOE can be an important tool to improve health care, but its effectiveness in improving the outcomes of critically ill patients is unproven and may require ICU-specific reengineering and customization to achieve the goal of a demonstrable mortality reduction.

## Key messages

• Implementation of Computerized physician order entry (CPOE) was found to be associated with no significant changes in morbidity and mortality of critically ill patients in the immediate period and up to 12 months afterwards.

• These findings were also seen in four high risk groups of patients: age 12-17 years, traumatic brain injury, diagnosis of sepsis on admission and admission APACHE II ≥ 23.

• CPOE effectiveness in the ICU may require ICU-specific reengineering and customization to achieve the goal of mortality reduction.

## Competing interests

The authors declare that they have no competing interests.

## Authors' contributions

**HD: **Study conception and design, data analysis, interpretation of data, drafting the manuscript

**HT: **Acquisition of data, data analysis, manuscript revision

**AC: **Study conception, manuscript revision

**MH: **Study conception, interpretation of data, manuscript revision

**ST: **Study conception, interpretation of data, manuscript revision

**YA: **Study conception and design, interpretation of data analysis, drafting the manuscript

All authors made critical revisions and approved the final manuscript.

## Pre-publication history

The pre-publication history for this paper can be accessed here:

http://www.biomedcentral.com/1472-6947/11/71/prepub
